# Writing for publication: institutional support provides an enabling environment

**DOI:** 10.1186/s12909-016-0642-0

**Published:** 2016-04-18

**Authors:** Beverley Kramer, Elena Libhaber

**Affiliations:** Health Sciences Research Office, Faculty of Health Sciences, University of Witwatersrand, Johannesburg, South Africa

**Keywords:** Writing workshops, Writing retreats, Writing skills, Scientific writing

## Abstract

**Background:**

Due to the excessive service delivery loads in public hospitals supported by academic institutions in developing environments, researchers at these institutions have little time to develop scientific writing skills or to write up their research. It is imperative to expand the writing skills of researchers and train the next generation of health sciences academics in order to disseminate research findings. This study reports on the implementation of approaches for writing and publication and the extent of support to staff suffering from the overload of service delivery and of heavy teaching duties.

**Methods:**

Workshops in scientific writing and writing retreats were initiated and were offered to all staff.

**Results:**

Feedback from participants of the writing skills workshops indicated that the workshops provided an injection of confidence and proficiency. Protected writing time resulted in 132 papers submitted to journals and 95 in preparation from 230 participants of the writing retreats over a two year period. Staff commended the off-site, collegial environment, which also supported future collaboration with new-found colleagues.

**Conclusion:**

This enabling environment facilitates not only the development of writing skills per se, but also the dissemination of the generated scientific knowledge. In addition, the training in writing skills of this generation will be of value in the training of future cohorts in countries with similar health care deliverables.

## Background

Dissemination of research and growing the knowledge base in health sciences is important for patient management and health policy development. Scientific writing in the health sciences underpins research, as results of research are of little use if they are not distributed for peer-review and implementation. Thus, the ability for health scientists to write, and to have the time to write, is important in translating findings into scientific literature. Yet many academics agree that writing for publication is one of the most difficult aspects of the process of research.

In the more recent past there has been increased pressure to publish [1], not only on the individual, but also from an institutional perspective [2, 3]. However, South Africa is faced with a crippling quadruple burden of disease [4], which results in excessive levels of service delivery for clinicians based at local institutions. More specifically in Gauteng province where our institution is based, high levels of HIV/AIDS [5] add to clinical loads. Clinicians thus do not have the necessary time to write up their research due to the pressure in the public hospitals [6].

As a proportion of institutional funding may be derived from research outputs, there has been a need for increased productivity at Universities [7] in order to generate income. At our Institution too, funding is received from the South African Department of Higher Education and Training (DHET) for research published in journals “accredited” by the DHET. Besides the funding, publications produced by institutions are important to illustrate the nature of research being undertaken and the development of a particular field or area of expertise [1]. This will usually bring prestige to the University [8]. Publication is of importance too, to the individual for career advancement [7] and in some cases for remuneration.

Sharma [9] maintains that “medical writing is both a science and an art” and for some, many barriers to writing have been identified. It appears that often medical professionals are reluctant to write [7, 10]. In order to write, both the knowledge of one’s subject and the aptitude to write are required. Barriers to writing are said to include the lack of self-confidence and difficulty with [1, 11, 12], anxiety of failing [1, 3, 13], finding writing to be intimidating and having writers block [14]. Lack of time [1, 11, 12], interrupted time [15] and workload [1, 11, 12] are often identified as major obstacles to writing. In addition, administrative activities are seen as a major barrier as they limit time for staff to follow scholarly activities [16]. The “opportunity to work on a single task” [15] or having uninterrupted (protected) time, is a solution which is particularly noteworthy.

In a developing third world country with a heavy burden of disease such as that in South Africa, producing research and the dissemination of this research through publication is additionally important in training our next generation of health science professionals and leaders. As globalisation continues to impact health, health care and research infrastructure [17], it is important that we develop the relevant skills within our researchers in order to expand our capacity. Support for these skills requires financial assistance.

A variety of courses [18] or workshops [18] and other implementations are described in the literature to develop writing skills. Writing retreats [19, 20], writing groups [21–23] and collaborative writing [7] appear in the more recent literature, while combined writing courses and writing groups [3, 13] are also documented. However at our Institution we did not only wish to improve writing skills, but wished to establish a community of practice [24] as communicative skills are said to be improved through practice, reflection and critique. [25] In addition, as Badenhorst [26] argues that “writing begets writing”, we wished to provide protected time in the busy schedules of our academics (including clinicians) in order for them to achieve their writing. Like Castle and Keane [14] we also believed that the writing environment or space where the writing occurs, is important.

In the case of scientific writing for health professionals, publications by the nursing fraternity appear most often in the literature [1, 3, 8, 27–29]. While these experiences are helpful, there is little in the literature directed at other health sciences professionals and especially at medical doctors who generally have very limited time for writing.

The most important factors in facilitating publication, besides the scientific content of the article, appear to be a combination of good writing skills and commitment of time. Although resources at our Institution were restricted, the University of the Witwatersrand (Wits) Faculty of Health Sciences Research Office (HSRO) recognized the need to provide academics with institutional aid in writing skills through workshops. In addition, the HSRO attempted to ring-fence time through writing “retreats” and to provide a supportive/nurturing environment to facilitate the writing of papers. This paper provides background on the creation, operation, financial costs and outcomes in setting up writing skills workshops and writing retreats in our Institution where there are large differences in needs and skills. This would assist not only the postgraduate students and staff, but also the long-term goal of the Institution to increase and disseminate its research output.

All the data utilized in this study emanated from the Faculty of Health Sciences, University of the Witwatersrand. Permission to utilize de-identified comments and data recorded from the staff/student evaluations of writing retreats, workshops and courses was provided by the Human Ethics Research Committee of the University of the Witwatersrand (M140756).

## Methods

### The institution

The Wits Faculty of Health Sciences is situated in Johannesburg, which is the centre of the commercial and industrial hub of South Africa. The Health Sciences Institution trains both undergraduate and postgraduate students (local and international) in all health sciences disciplines. It is responsible for clinical training and patient care at three large academic hospital teaching platforms which service approximately 2000 patient beds. There is thus a huge service delivery component in the already busy academic activities of the majority of our staff. In recent years many of our staff and students have been presenting without the necessary writing skills or English language skills [14].

### Workshops in scientific writing

A number of workshops were initiated as early as 2007. From 2008, writing workshops were consolidated and offered by the HSRO. Highly experienced senior researchers were utilized in the provision of these workshops which included topics such as writing a literature review, writing a thesis and writing for scientific publication. In addition, short tutorials on specific aspects of an article were provided, such as the “abstract”, “introduction”, “discussion” and so on. Special annual workshops on writing skills which included critique of articles were introduced in 2011 and were provided by staff and invited international researchers. In addition, and as funding allowed, one-on-one mentoring in writing was provided for postgraduate students.

Attendance at workshops varied from 20 to 25, while the short tutorials were attended by between 7–25 individuals. Attendees were mainly postgraduate students (67 % were Masters students and 33 % Ph.Ds) and researchers, many of whom were based in the clinical disciplines (49 %).

### Initiation of writing retreats

It was envisaged that due to the teaching loads, heavy service delivery and administrative duties of the staff, protected time could facilitate the writing of articles. Hence an HSRO-facilitated small group writing retreat was introduced in 2010 and repeated in 2011. The outcome of the writing retreat was expected to be the production of an article and submission of the article to a journal. The retreats were “residential” at one of the off-site facilities belonging to the Institution. The group in each case was confined to 8–10 staff per retreat and two facilitators. The two facilitators were highly skilled senior academics with PhDs and expertise in scientific writing. The facilitators read and provided comments on the writing during the retreat. The retreat consisted of a three day stay at a distant site. Distance was thought to be important in order to provide separation from the pressure of work and family. Funding of these retreats cost $1 900 (2010) and $2 600 (2011) which included the cost of two facilitators, travel to the distant site, accommodation and meals at each retreat.

The venue where writing occurred was a conference room with adequate space for the academics. Each academic brought their own computer, while internet facilities, and a printer and paper were provided. “Selection” of the participants was made on the basis of completed data collection and analysis, and that the participant was in the writing-up phase of their article. A small “community of scholars” from diverse disciplines was thus set up. It is important to note that these retreats were not methodological in nature, but were protected writing periods to allow for completion of an article.

In 2012, due to the continued request for “time to write” by academics, the writing retreat construction was expanded. This was supported by an increase in funding for this activity from the Institution’s Strategic Planning and Allocation of Resources Committee (SPARC) Fund which totaled $36 400. The call for applications was announced and included the support of three types of writing retreat:

- short (2–3 day) writing retreats which were to be facilitated by mentors (senior staff with publication records), for those who required assistance with writing;
- short (2–3 day) writing retreats which were not facilitated by mentors. This for academics who were already skilled and did not perceive the need for additional assistance with writing; and
- “spaced-day” writing retreats, non-facilitated, for those academics who did not wish to be away from their home environment for periods of time and who preferred not to have concentrated blocks for writing.

In 2013 and 2014, while funding diminished, the HSRO was once again able to provide writing retreats in the format used in 2012. The total cost of the 2013 writing retreats was $29 200 and for 2014, it was $15 500.

Generally an environment which would enhance scholarship, dialogue and the development of a community of scholars was sought. These were conference facilities off-site of the Institution. The different groups varied in number, facilitation or not, in the number of days and in the composition of the groups. However, the common focus of all the groups was the production of one paper per participant for publication. Participants were from diverse disciplines, ages, sex and experience in writing.

## Results

### Outcomes of writing retreats

In 2010 and 2011 the two small retreats resulted in eight submissions each. The retreat in 2012 resulted in 186 submissions from 18 groups, of which 24 publications emanated from a School where there had been little publication in previous years.

Fourteen groups were funded in the round of retreats in 2013. From these groups 92 articles were submitted. In 2014, 12 groups were funded and 38 articles emanated.

### Funding

In order to determine whether there was a financial benefit to the University through the increased publication of articles by means of the writing retreats, we calculated the financial cost of writing a single paper. In 2012 the cost of writing 186 articles was $36 400, in 2013 total submissions were 92 at a cost of $29 200 and in 2014, $15 500 resulted in 40 submissions. Thus a total of 318 submissions were made at a total cost of $81 100 over the three years (Note: this does not include the costs of the research). Therefore the cost of generating an article in a collegial environment at a writing retreat was $255. The University receives subsidy of $11000 per publication. Thus, even if only half of the above papers (159) are finally published, the financial benefit to the University could be in the region of approximately $1, 75 million.

### Appraisal of workshops

Assessment of the writing courses produced positive feedback such as “as a young scientist I was inspired to think and to write. I left with the feeling that writing papers is possible and not as much a huge monster as it seems.” In addition a further comment from a participant was “really inspirational and really good direction and understanding of purposes of each writing section and sub-section.” Young staff felt that it was a “very helpful guide to this wilderness of writing” and “instilled confidence.”

As many of those attending the writing skills workshops were postgraduate students and emergent researchers, training in this aspect of research was deemed of benefit from the comments of the participants. This aspect of research training should be of lasting value not only to the authors, but also to the Institution. As with Murray and Newton [30], our researchers commented on the benefit of the courses, but recommended ongoing support.

## Discussion

The cost of providing protected time/writing retreats for staff to write articles was found to be relatively low compared to the subsidy gained from publication and is thus advantageous to the institution. Unfortunately comparative studies in the literature on similar costs were not found.

Different approaches to enhancing writing for publication as described in the literature are “short stops” which use time as a group for an hour every 4–6 weeks [16] and “collaborative” writing groups [1], while workshops and a series of peer writing groups and independent study were found to promote scholarly writing among staff [13]. We preferred to develop both writing workshops and protected time for writing. Generally, time for writing appears to be the major impediment for success [13].

Important in the development of writing is the advancement of a community of scholars and a pleasant writing environment [19].

A limitation of this study is that a control group was not utilised. Thus, it is not known if the participants would have been equally successful at producing papers without the retreats (see also [13, 21]). However, the achievement of the submission of a publication by the majority of the participants was impressive following such a short writing period.

Many of our writing retreats took place off-site of our Institution in pleasant and comfortable surroundings, away from the pressures and telephones at work. Removal of the writers from their place of work has been shown to reduce the anxiety associated with producing scholarly publications [19]. While the participants return to the pressures of their jobs following the writing retreat, it is important to provide ongoing opportunities for writing [14].

The HSRO found the writing retreats to be productive both from the perspective of allowing staff to complete the writing of an article, and from the perspective of augmenting staff output and dissemination of research. In order to allow staff to pursue quiet and protected time for writing, the HSRO has set up a new “writing room” in an environment removed from teaching and clinical activities. The importance of a supportive environment for staff has been noted by several authors [19, 21].

The staff valued the commitment of the HSRO in providing time, money and pleasant surroundings for writing of articles. The retreat was said to be “constructive and highly beneficial”. These retreats not only led to the submission of publications, but also to the development of collegial relationships between disparate academics. As a participant mentioned “I found it very helpful to be with a group who are from different academic fields as I was exposed to different approaches. I formed new relationships which I am hoping will result in collaborations of some sort in the future.” We believe these experiences are of value as Jackson [31] maintains that organisational life can often impede collegial relationships.

According to the CenterWatch analysis [9], the medical writing market has doubled in size in the last five years. As the development of medical science requires the clear presentation of data for perusal and criticism by other scientists, it is incumbent on health science institutions to provide the necessary training and development of skills in health science professionals.

In order to equip our staff with the relevant skills to undertake research, to analyse the data and write up articles, our Faculty of Health Sciences has put in place a variety of training modules in research methodology and in biostatistics [6] which are proving to be useful to the staff. The extent of the support (multiple courses on offer at different times of the week and day, one-on-one tutorials, multiple writing workshops), is seen as being a model for other institutions in countries with high burdens of disease.

## Conclusion

Protected time for writing was of benefit to participants. From our analysis, the cost of providing staff with protected time to generate their research articles is relatively low. The benefit of publishing a manuscript is of huge importance in disseminating scientific findings which would otherwise be lost or confined to the laboratory where it originated. In addition, publication is supportive of the Institution in attaining its research goals and deriving funding which would continue in a reiterative process to provide finances for continuing research productivity.

As many of those attending the writing skills workshops were postgraduate students and emergent researchers, training in this aspect of research was deemed of benefit from the comments of the participants. The training of emerging researchers in scientific writing skills will lead to improved skills in the next generation of health sciences professionals.

## Consent to publish statements

Permission to utilize de-identified comments and data recorded from the staff/student evaluations of writing retreats, workshops and courses was provided by the Human Ethics Research Committee of the University of the Witwatersrand (M140756).

## Availability of data and materials statement

All the data utilized in this study emanated from the Faculty of Health Sciences, University of the Witwatersrand.
